# *DDX58* and Classic Singleton-Merten Syndrome

**DOI:** 10.1007/s10875-018-0572-1

**Published:** 2018-12-20

**Authors:** Carlos R. Ferreira, Yanick J. Crow, William A. Gahl, Pamela J. Gardner, Raphaela Goldbach-Mansky, Sun Hur, Adriana Almeida de Jesús, Michele Nehrebecky, Ji Woo Park, Tracy A. Briggs

**Affiliations:** 1 Medical Genetics Branch, National Human Genome Research Institute, National Institutes of Health, Bethesda, MD USA; 2 Institute of Genetics and Molecular Medicine, Centre for Genomic and Experimental Medicine, The University of Edinburgh, Edinburgh, UK; 3 Laboratory of Neurogenetics and Neuroinflammation, Paris Descartes University, Sorbonne-Paris-Cité, Institut Imagine, Paris, France; 4 Office of the Clinical Director and Medical Genetics Branch, National Human Genome Research Institute, National Institutes of Health, Bethesda, MD USA; 5 Office of the Clinical Director, National Institute of Dental and Craniofacial Research, National Institutes of Health, Bethesda, MD USA; 6 Translational Autoinflammatory Disease Studies (TADS), National Institute of Allergy and Infectious Diseases (NIAID) National Institutes of Health, Bethesda, MD USA; 7 Biological Chemistry and Molecular Pharmacology, Harvard Medical School, Boston, USA; 8 Biology Department in Morrissey College of Arts and Sciences, Boston College, Chestnut Hill, USA; 9 Manchester Centre for Genomic Medicine, St Mary’s Hospital, Manchester University Hospitals NHS Foundation Trust Manchester Academic Health Sciences Centre, Manchester, UK; 10 Division of Evolution and Genomic Sciences, School of Biological Sciences, University of Manchester, Manchester, UK

**Keywords:** Interferonopathy, retinoic acid-inducible gene I, Singleton-Merten syndrome, type I interferon

## Abstract

**Purpose:**

Singleton-Merten syndrome manifests as dental dysplasia, glaucoma, psoriasis, aortic calcification, and skeletal abnormalities including tendon rupture and arthropathy. Pathogenic variants in *IFIH1* have previously been associated with the classic Singleton-Merten syndrome, while variants in *DDX58* has been described in association with a milder phenotype, which is suggested to have a better prognosis. We studied a family with severe, “classic” Singleton-Merten syndrome.

**Methods:**

We undertook clinical phenotyping, next-generation sequencing, and functional studies of type I interferon production in patient whole blood and assessed the type I interferon promoter activity in HEK293 cells transfected with wild-type or mutant *DDX58* stimulated with Poly I:C.

**Results:**

We demonstrate a *DDX58* autosomal dominant gain-of-function mutation, with constitutive upregulation of type I interferon.

**Conclusions:**

*DDX58* mutations may be associated with the classic features of Singleton-Merten syndrome including dental dysplasia, tendon rupture, and severe cardiac sequela.

## Introduction

Singleton-Merten syndrome (SMS [MIM 182250]) is an autosomal dominant disorder, encompassing the cardinal features of dental dysplasia, glaucoma, psoriasis, aortic calcification, and skeletal abnormalities, including osteoporosis, contractures, and tendon rupture. In five SMS patients from three families, Rutsch et al. [1] described a heterozygous missense pathogenic variant, c.2465G>A (p.Arg822Gln), in interferon-induced with helicase C domain 1 (*IFIH1*) as causative. *IFIH1* encodes the cytosolic double-stranded RNA sensor melanoma differentiation-associated protein 5 (MDA5), a member of the RIG-I-like receptor (RLR) family and an integral sensor in the type I interferon pathway. Pathogenic variants in *IFIH1* had previously been associated with a variety of neuroinflammatory phenotypes including Aicardi-Goutières syndrome (AGS) and hereditary spastic paraparesis [2, 3]. Functional investigations in both the SMS and AGS studies demonstrated that the observed heterozygous *IFIH1* pathogenic variants resulted in a gain-of-function with induction of type I interferon production and increased expression of interferon-stimulated genes (ISGs) [1–3]. Subsequently, we reported additional families that expand the *IFIH1*-associated disease overlapping phenotype, with features of chilblains, intracranial calcification, and neurological sequela consistent with an AGS diagnosis, and psoriasis, dental dysplasia, and contractures in keeping with SMS within the same families [4, 5].

Jang et al. [6] then reported pathogenic variants in *DDX58*, encoding another cytosolic double-stranded RNA sensor and member of the RLR family, *DDX58* or retinoic-acid-inducible gene I (RIG-I). Specifically, they described heterozygous pathogenic variants in two families with multiple members who manifested variable features of SMS including psoriasis, acro-osteolysis, and glaucoma; aortic and valvular calcification were also prominent in the first family. Dental dysplasia was not observed in either family, nor was tendon rupture, and the disease phenotype was considered to be milder and associated with a better prognosis than classic SMS. Consistent with the role of RIG-I in RNA sensing and type I interferon induction, pathogenic variants were shown to result in constitutive interferon activation.

We now report a Caucasian family with characteristic features of SMS secondary to a *DDX58* heterozygous gain-of-function variant.

## Methods

Both patients were enrolled in clinical protocol 76-HG-0238 (identifier: NCT00369421), approved by the NHGRI Institutional Review Board, and gave written informed consent. Whole exome sequencing was undertaken using SeqCap EZ Exome+UTR Library using a HiSeq 2500 (Illumina). Alignment, genotype calling, and annotation were undertaken using the Illumina aligner “ELAND” (Efficient Large-scale Alignment of Nucleotide Databases), Samtools, and Annovar. Sanger confirmation was undertaken of the *DDX58* variant. Primer sequences are available on request.

Structural analysis was undertaken based on the structure of RIG-I [7, 8]. RIG-I wild-type and mutant plasmid constructs were devised and transiently co-transfected with an IFN-b promoter-driven firefly luciferase reporter plasmid into a HEK293 cell line. At 6 h, post-transfection cells were transfected with in vitro–transcribed dsRNA (poly I:C 0.5 mg), and at 24 h, post-stimulation cells were lysed and IFN-b promoter activity assayed, as per Peisley et al. [9]. Significant *P* values were calculated using a one-tailed, unpaired *t* test, comparing mutant IFN production with WT RIG-I.

Gene expression of selected interferon-stimulated genes (ISGs) was determined by Nanostring (NanoString Technologies, Seattle, WA) and an IFN-score was calculated. Standardized interferon score is the sum of 25 Nanostring counts that were standardized by subtracting the mean of healthy controls and dividing by standard deviation of the healthy controls. Means and SDs of the IFN score are depicted in parenthesis for each group of individuals, as per Kim et al. [10].

## Results

### Case Histories

**Fig. 1:**
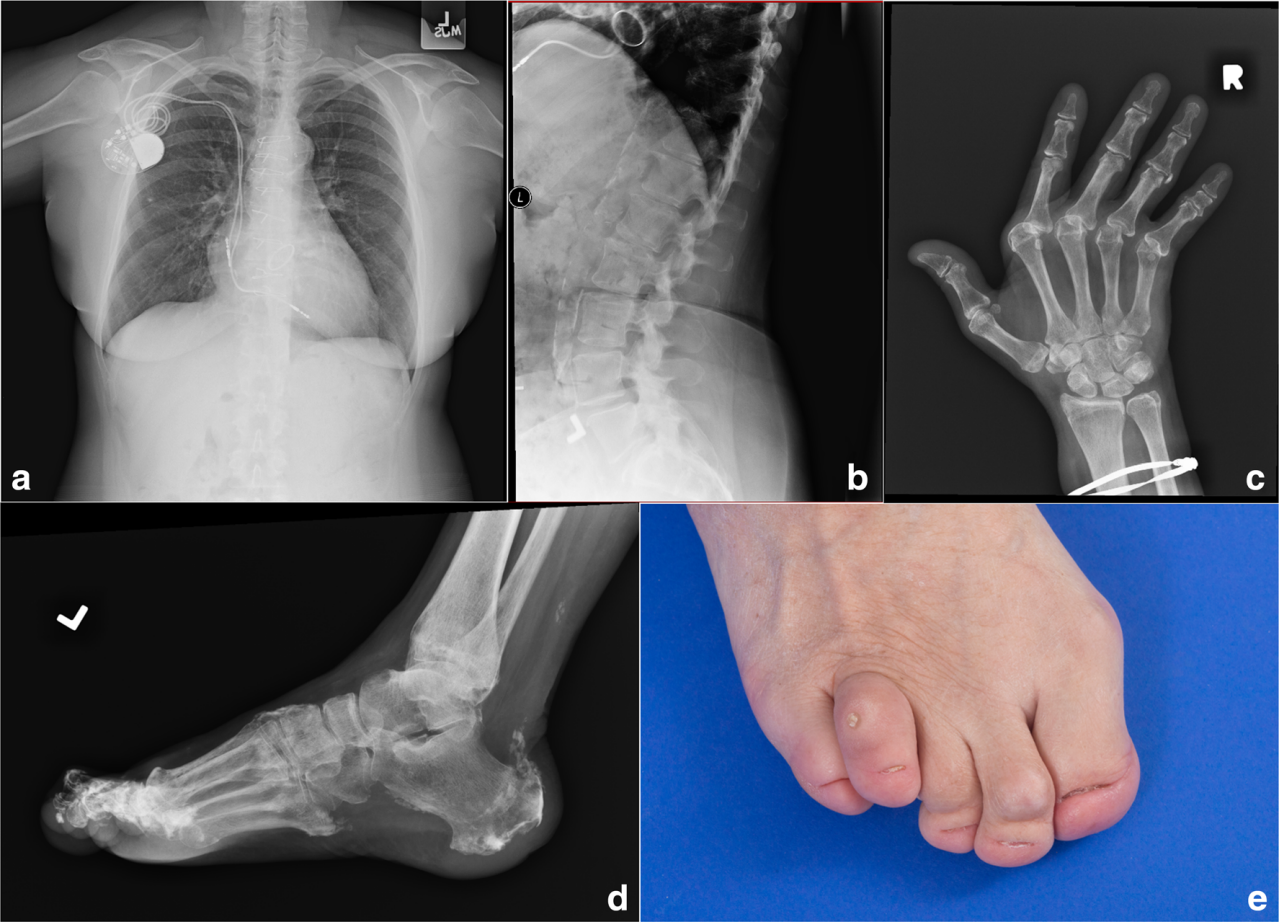
Clinical manifestations of SMS in the family. **a** A pacemaker generator, sternotomy wires, and a prosthetic mitral valve are present. **b** Calcification of the abdominal aorta is observed and the lumbar vertebral bodies show low bone mineral density. **c** Joint deformities are evident due to metacarpophalangeal joint subluxation. **d** Calcification is seen at the insertion of the Achilles tendon and the plantar fascia. **e** Toenails are hypoplastic and subcutaneous calcium deposition is evident over the fourth toe

The proband presented at birth with markedly hypoplastic/aplastic toenails and scalp psoriasis, which resolved spontaneously within 12 months. At age 3 years, she developed glaucoma requiring seven surgical procedures. She demonstrated delayed eruption of her secondary dentition with the absence of many secondary teeth at age 15 years; she then began to lose her secondary dentition and required a full set of dentures by her early twenties. In her teens, widespread psoriasis recurred for over a decade. She suffered a right Achilles tendon rupture at age 26 years, a rupture of the left thumb tendon in her late thirties, and bilateral avulsions of the quadriceps tendons from the patellae at age 44 years. In her late forties, she developed features consistent with Jaccoud arthropathy. Skeletal radiographs showed bilateral calcification of the quadriceps tendons and tibial tubercles, and DXA scans demonstrated osteopenia (T-score, − 2). At age 42 years, she was diagnosed with bilateral cataracts. Cardiac disease was a prominent feature from the age of 34 years, with chest pain and dyspnea caused by aortic valve calcification, eventually necessitating aortic valve replacement. At 43 years of age, third-degree AV block led to the placement of a dual-chamber pacemaker, and at age 52 years, mechanical mitral valve replacement was undertaken due to severe calcification (Fig. 1a); a single-vessel CABG was performed to relieve a 50% occlusion of the right coronary artery. A chest radiograph at this time revealed a tortuous and calcified thoracic aorta. Upon evaluation at age 59 years, she had marked calcification of the abdominal aorta (Fig. 1b), ulnar deviation of the fingers (Fig. 1c), and calcification of the Achilles tendons and plantar fascia (Fig. 1d). At age 60 years, she died from cardiac disease. Autopsy demonstrated extensive calcification of myocardium and papillary muscles and large arterial medial calcification of the aorta and carotid arteries, with superimposed coronary atherosclerosis and acute myocardial infarction of the interventricular septum.

The proband’s 32-year-old son exhibited similar features, with hypoplastic/aplastic toenails from birth (Fig. 1e), spontaneously resolving psoriasis in childhood, and bilateral glaucoma requiring surgery at age 5 years.

**Fig. 2:**
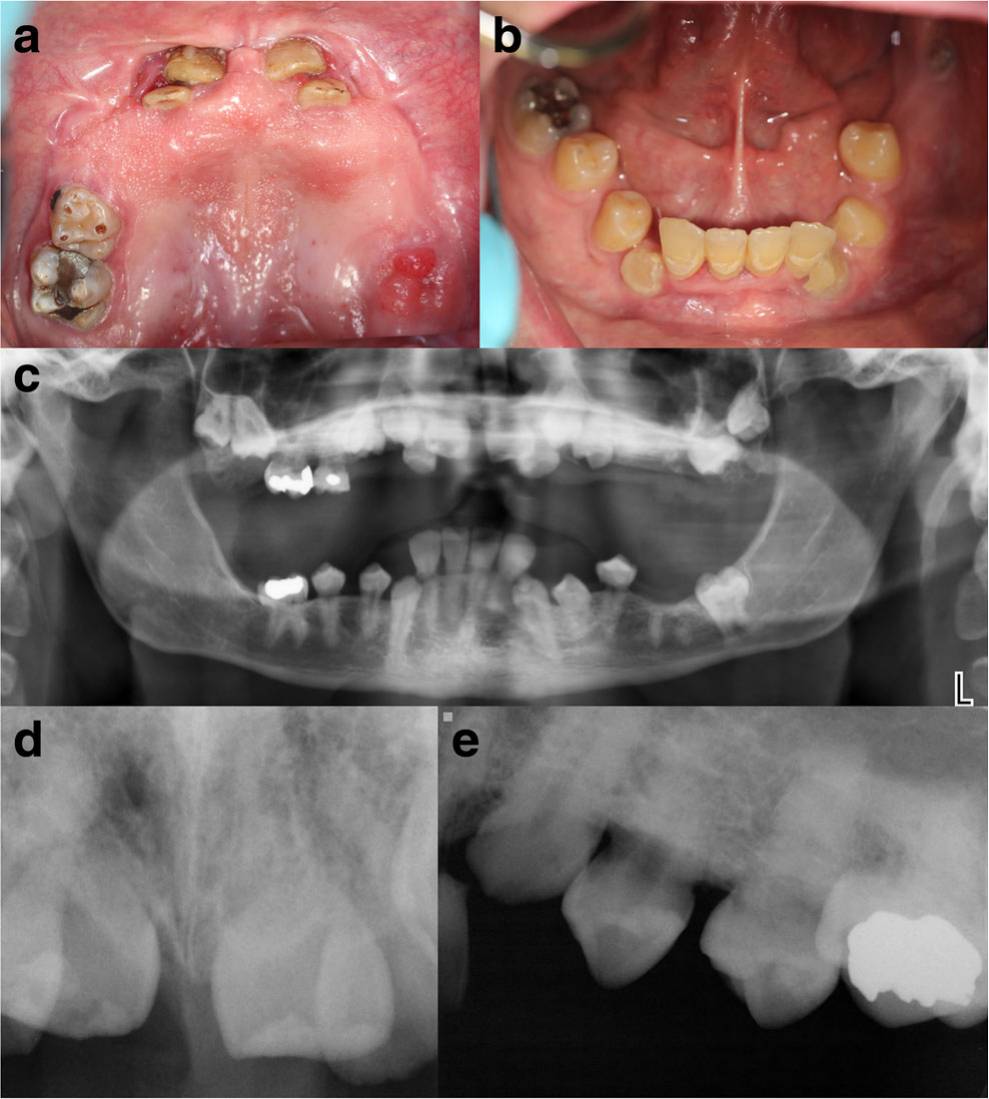
Dental manifestations of SMS. Clinical photography of maxillary **a** and mandibular **b** teeth showing failure of eruption of many permanent teeth. A dental panoramic radiograph **c** confirmed that most of these permanent teeth were impacted in the bone. **d** Anterior periapical radiograph showing very short roots. **e** Periapical view of upper left quadrant teeth revealing absent periodontal ligament space consistent with ankylosis

Despite having a normal eruption of the primary teeth, there was a failure to exfoliate naturally. The primary lower central and lateral incisors were extracted when he was a young child, and the permanent teeth erupted normally. Other primary teeth were extracted much later after root development had ceased, resulting in a failure of many permanent teeth to erupt into occlusion. Orthodontic therapy failed to pull any of the teeth into occlusion. He has worn a full upper denture over the partially erupted teeth since the age of 15 years (Fig. 2a–b).

Clinically, the teeth visible in the oral cavity were at various stages of eruption. There was one retained upper primary molar that was not mobile. There was a striking lack of alveolar bone height in both arches despite the eruption of multiple permanent teeth in the mandible. Dental decay affected the teeth under the upper denture, but there was very little gingival inflammation or gingival recession to suggest periodontal disease.

Radiographically, there appeared to be a normal number of developing permanent teeth, most of which remained subgingival and partially impacted in the bone (Fig. 2c). Tooth crown development appeared normal; however, the roots were very short (Fig. 2d) and tapered with closed root apices. While the pulp chamber was visible to suggest the submerged teeth were vital, the periodontal ligament space could not be discerned (Fig. 2e) consistent with root ankylosis. The failure of normal permanent tooth eruption would result in the reduction in height of the alveolar bone.

He suffered tendon ruptures in the hands in his late teens, which resulted in progressive deformities from age 20 years. He was first recognized to have aortic calcification in his early twenties. Neither the proband nor her son demonstrated any neurological manifestations, and CT scans in adulthood did not show intracranial calcification.

In the extended family history, the proband’s mother lost all secondary dentition in her 20s, while a maternal niece was reported to have hypoplastic nails.

### Genetics

Whole exome sequencing in both mother and son revealed a heterozygous *DDX58* variant NM_014314.3: c.1551G>C (p.Gln517His), confirmed by Sanger sequencing. This variant was novel, not observed in over 245,000 alleles in gnomAD. The mutated amino acid is located adjacent to the RNA phosphate backbone of the protein.

### Functional Studies

**Fig. 3:**
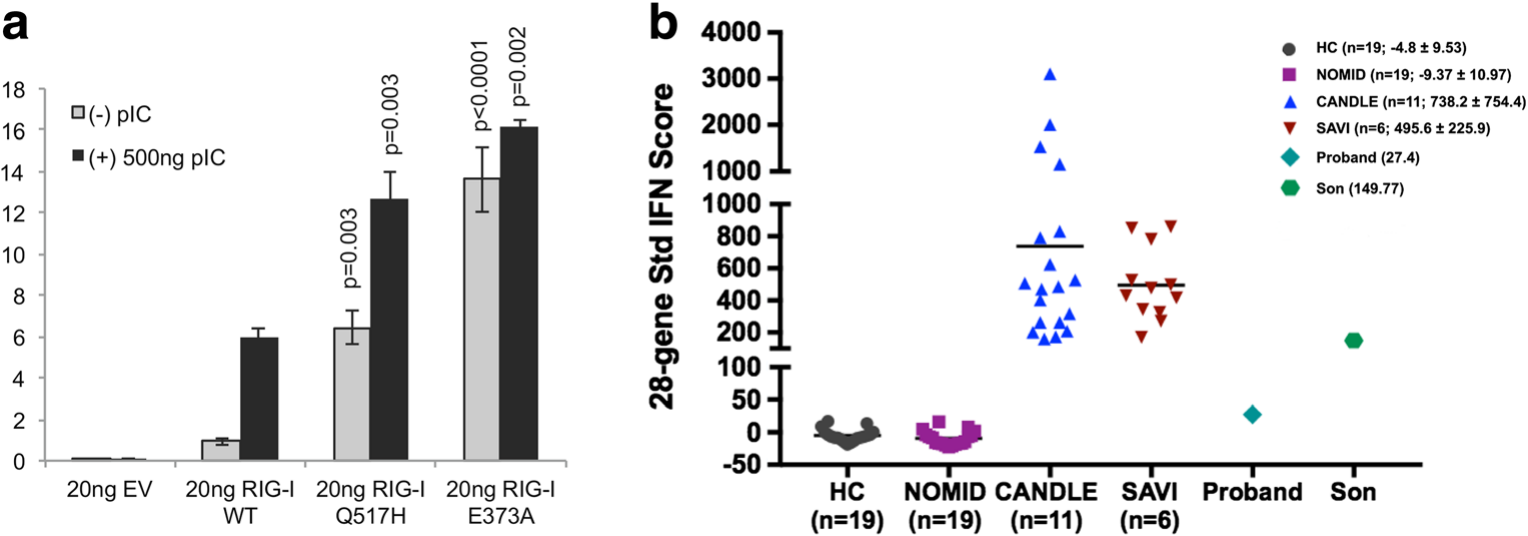
Type I interferon induction is associated with a Gln517His heterozygous *DDX58* variant. **a** Interferon-beta luciferase assay following transient transfection of HEK293 cells demonstrated significantly elevated basal and poly I:C stimulated interferon induction in both the Gln517His mutant and the previously described Glu373Ala mutant relative to WT expression. **b** Gene expression of selected 28 interferon-stimulated genes (ISGs) was assayed, and an IFN-score was calculated. Mean and SD of the IFN score are depicted in parenthesis for each group of individuals. HC: healthy controls; NOMID: neonatal-onset multisystem inflammatory disease; CANDLE: chronic atypical neutrophilic dermatosis with lipodystrophy and elevated temperature; SAVI: STING-associated vasculopathy with onset in infancy; 4088: proband’s son; 4089: proband

A type I interferon assay was performed following transient transfection of wild-type RIG-I, the Gln517His mutant, or the previously described Glu373Ala mutant [9]. Both mutant constructs demonstrated significantly elevated basal interferon induction relative to WT expression (Fig. 3a). Furthermore, significantly enhanced type I interferon induction was observed following dsRNA stimulation in both mutant constructs compared to wild type.

Further support for a RIG-I gain-of-function in association with the Gln517His mutant was demonstrated by upregulation of ISGs in whole blood in the son’s sample, though not the probands (Fig. 3b).

## Discussion

Here, we demonstrate that a pathogenic heterozygous variant in *DDX58* can manifest as classical SMS, which includes both a dental and tendon rupture phenotype, not described in the previous report by Jang et al. [6]. The severity of the multi-systemic manifestations resulted in early-onset morbidity in both the proband and her son and was responsible for mortality in the proband secondary to cardiac sequelae. We, therefore, conclude that *DDX58* gain-of-function variants may present with a phenotype as severe and diverse as that previously described in *IFIH1*-associated SMS [1, 11] and that there is variability within those patients with *DDX58* pathogenic mutations [6], as shown in Table 1.

**Table 1:** Phenotypic manifestations of clinically classified SMS associated with causative *DDX58* and *IFIH1* variants

	Total *n* (%) from this study	Total *n* (%) reported with *DDX58*-associated SMS [6]	Total *n* (%) reported with *IFIH1*-associated SMS [1, 11]
Glaucoma	2/2 (100)	10/11 (91)	5/10 (50)
Short stature	0/2 (0)	2/11 (18)	6/9 (67)
Aortic and valvular calcification	2/2 (100)	5/7 (71)	0/11 (91)
Cardiac arrhythmia	1/2 (50)	0/11(0)	6/11 (55)
Acro-osteolysis or tuft erosion of distal phalanx	0/2 (0)	8/8 (100)	6/9 (67)
Wide medullary cavities in the phalanges	0/2 (0)	0/11 (0)	9/10 (90)
Subungual calcifications	0/2 (0)	0/11 (0)	3/8 (38)
Tendon rupture	2/2 (100)	0/11 (0)	6/11(55)
Joint subluxation	2/2 (100)	1/11 (9)	8/9 (89)
Thick neurocranium	0/2 (0)	0/11 (0)	8/9 (89)
Scoliosis	0/2 (0)	0/11 (0)	3/10 (30)
Dental problems	2/2 (100)	0/11 (0)	10/11 (91)
Dysmorphic facies	0/2 (0)	0/11 (0)	7/7 (100)
Weakness/hypotonia	0/2 (0)	0/11 (0)	8/10 (80)
Psoriasiform rash	2/2 (100)	7/11 (64)	8/9 (89)

In addition to the marked clinical overlap between *IFIH1* and *DDX58*, we also acknowledge similarities to other described monogenic type I interferon disorders. For example, glaucoma is observed in AGS beyond *IFIH1*, most commonly in association with *SAMHD1*-associated disease [12]; arthropathy is a feature of a number of type I interferonopathies including the recently described DNASEII deficiency [13]; finally, we have recently noted early loss of secondary dentition in an adult with a pathogenic homozygous *RNASEH2B* variant and in several patients with CANDLE (unpublished). Of particular interest, given that IFIH1 has been associated with both AGS and SMS and that we have reported overlapping patients previously [4, 5], neurological manifestations associated with *DDX58* mutations are yet to be reported and the cranial imaging in our family was normal.

Our clinical and functional studies support previous findings that *DDX58* gain-of-function pathogenic variants are associated with aberrant interferon production. Whether the normal score at the time of the blood draw in the proband reflects lower systemic inflammation over time, or is part of a variation with intermittent systemic flares, could not be assessed because no further samples were available. We note that not all cases of monogenic interferonopathy manifest an elevated ISG and indeed, 31% of cases of *RNASEH2B*-associated AGS did not have an elevated ISG on testing [12].

Making a genetic diagnosis in such families and understanding the full phenotypic spectrum are important in terms of tailored management and screening, as well as allowing provision of genetic testing, including prenatal testing to at-risk relatives. In type I interferon-driven disorders, diagnosis is also important because targeted anti-interferon therapies, such as JAK-STAT inhibition, and anti-IFN receptor antibodies hold the promise of personalized treatment directed toward the underlying genetic etiology [14–16].
